# An Optimization Routing Algorithm for Green Communication in Underground Mines

**DOI:** 10.3390/s18061950

**Published:** 2018-06-15

**Authors:** Heng Xu, Qiyue Li, Jianping Wang, Guojun Luo, Chenghui Zhu, Wei Sun

**Affiliations:** School of Electrical Engineering and Automation, Hefei University of Technology, Hefei 230009, China; neosonny@163.com (H.X.); liqiyue@mail.ustc.edu.cn (Q.L.); wjphfut@126.com (J.W.); hfutgjluo@163.com (G.L.); hgdzdhzhuch@163.com (C.Z.)

**Keywords:** green communication, underground mine, DCMST, GA, heterogeneous network

## Abstract

With the long-term dependence of humans on ore-based energy, underground mines are utilized around the world, and underground mining is often dangerous. Therefore, many underground mines have established networks that manage and acquire information from sensor nodes deployed on miners and in other places. Since the power supplies of many mobile sensor nodes are batteries, green communication is an effective approach of reducing the energy consumption of a network and extending its longevity. To reduce the energy consumption of networks, all factors that negatively influence the lifetime should be considered. The degree constraint minimum spanning tree (DCMST) is introduced in this study to consider all the heterogeneous factors and assign weights for the next step of the evaluation. Then, a genetic algorithm (GA) is introduced to cluster sensor nodes in the network and balance energy consumption according to several heterogeneous factors and routing paths from DCMST. Based on a comparison of the simulation results, the optimization routing algorithm proposed in this study for use in green communication in underground mines can effectively reduce the network energy consumption and extend the lifetimes of networks.

## 1. Introduction

In a green communication underground mine (GCUM), installing mobile modules on the miners, as well as mobile equipment, requires numerous mobile nodes. Therefore, GCUMs are highly dynamic [1,2]. To track the locations of miners and perform other functions, it is important to obtain accurate data from mobile nodes. In this paper, the proposed technique will serve our program of localization in underground mines in this paper. Connectivity is essential between each mobile node in a network. Furthermore, connectivity and clustering techniques can be used to optimize the structure of a network with heterogeneous mobile nodes, but these methods increase the energy consumption of the network. To optimize green communication and the performance of the network and structure, reduce energy consumption and prolong the lifetime of the network, clustering algorithms are effective solutions for networks with large numbers of mobile nodes.

In a GCUM, batteries supply all the power to the mobile nodes. Battery supplement brings limited lifetime to these mobile nodes. The approach of green communication is introduced to save more energy to extend and lifespan of the network. Accidents are highly probable in underground mining [3]. In case of emergency or accident, the mobile node must use limited energy for communication with rescue forces. Therefore, green communication is important to save residual energy of mobile nodes and help to implement the functions in normal and emergency states. Anchor nodes that are fixed to the tunnel walls have wired power supplies and batteries for backup. However, long wires may have high costs and create risks since they can be damaged in accidents. Thus, anchor nodes are established to limit the energy consumption. In this paper, a routing algorithm is proposed to effectively reduce energy consumption from two perspectives. First, a degree constraint mine spanning tree (DCMST)-based algorithm is introduced to determine the lowest cost and most reliable routing paths. Second, a GA is introduced to optimize the cluster structure.

There are many tunnels, geological structures, and environmental factors that constitute the complex environment in underground mines. Such mines are often affected by unpredictable and extensive structural changes, especially when accidents occur. In such cases, the original communication paths are often disrupted. Therefore, in a GCUM, high connectivity between nodes is essential. In this paper, the DCMST algorithm is used to determine the lowest overhead option for routing paths and to optimize the quality of routing paths. In addition, based on the degree of nodes in the GCUM, nodes with high residual energy are established as cluster head nodes to balance energy consumption and extend the network lifetime.

Based on the former routing paths, nodes in the GCUM are clustered to balance the energy consumption of the green communication network. The cluster head nodes concurrently receive the data packets transmitted by all the cluster member nodes and receive the packets transmitted from other cluster head nodes or anchor nodes to each cluster member node. The timing and frequency of transmissions from the mobile nodes to the cluster head node will significantly affect the energy consumption of each node. If a mobile node exhausts its energy too early, it will lose all its data and impact the lifetime of the entire network. In addition, clustering algorithms can reduce the energy consumption caused by other factors.

Clustering algorithms are effective methods of improving the transmission of data in a network and reducing energy consumption. These algorithms can improve the data transmission capacity of mobile nodes and prevent nodes from becoming cluster head nodes that consume large amounts of energy. Furthermore, these optimization processes can prevent mobile nodes with low remaining energy levels from becoming cluster heads that consume large amounts of energy, thereby extending the lifetime of the network. In a GCUM, there are three types of nodes: anchor, multifunction, and original nodes. GAs have been applied to obtain optimal network solutions in many situations. GAs are characterized by high convergence and can be used in Wireless Sensor Networks (WSNs) with highly dynamic and multiple objectives to determine the optimal network cluster structure based on random searches.

However, most GAs applied in cluster analysis are applied to network environments without features. The parameters of nodes are often consistent in the network, and clustering algorithms are based on a single criterion. However, in a network with heterogeneous characteristics, if the nodes are clustered according to a single standard, although it may balance the energy consumption, it is not realistic green communication, and the overhead of the routing paths must be considered.

In a heterogeneous communication environment, it is relatively difficult to balance various heterogeneous factors [4,5]. Some optimization methods, such as DCMST, have been used in routing analysis to solve these problems. These algorithms can effectively handle issues associated with multiple heterogeneous factors, determine the optimal minimum overhead of the routing paths, and establish the roles of the mobile nodes according to their properties. The integrative capacity and flexibility of the GA can effectively solve dynamic and multiobjective structural optimization problems in heterogeneous networks. In this paper, the GCUM is a dynamic, multiobjective, and complex topological network. Thus, the DCMST is first applied to obtain the routing paths with the lowest overhead in the network. Then, the GA is applied to determine the optimal clustering structure.

The main contributions of the paper are as follows:

1. We develop a high throughput and low energy consumption routing path algorithm that preserves the connectivity and the degree the nodes are constrained, which is proposed to reduce the energy consumption of multipath communication and optimize green communication based on the DCMST.
2. According to the former routing paths, a GA is introduced to optimize the clustering process and balance the energy consumption and nodal loads of the network.
3. The routing algorithm proposed in this paper is based on the improved GA, which is applied in the heterogeneous nodal environment and optimizes the fitness function based on the weights of the routing paths from the DCMST. Finally, we optimized the energy consumption from many aspects to achieve the goal of green communication in the GCUM.

The remainder of this paper is organized as follows: Section 2 introduces the existing techniques for reducing the overhead in routing algorithms in green communication; Section 3 presents the node and energy consumption models; Section 4 introduces the method of guaranteed connectivity in the context of green communication based on the DCMST; Section 5 proposes a clustering technique to balance the energy consumption and nodal loads in a heterogeneous network based on the GA; The experiments and results are presented in Section 6; and Section 7 concludes the paper.

## 2. Related Work

The wireless sensor network (WSN) in underground mining is composed of a large number of sensor nodes that are deployed on miners or in other places. Thus, the WSN plays an important role in production and can save lives [6]. Simply summarizing the results of the paper [7], many instruments and equipment were invented by CRISO for various underground environments, e.g., high resolution, sub-surface radar for underground object and structure detection. In our research, the WSN is mainly used underground to achieve localization and other functions. Although WSN techniques are widely used in many fields, the WSN of green communication faces many challenges, especially in underground mining [8]. Wisam proposed a channel model for underground mine to reduce the energy consumption, noise, and electromagnetic interference [9]. Thus, green communication is significant for each network node to work long hours, and effective to maintain functionality, and achieve a long lifespan and better connectivity of the network [10].

The WSN in underground mining is used to collect information from sensor nodes that are deployed on miners and mobile equipment and at various locations that require supervision [11,12]. Numerous mobile sensor nodes are used in underground mining, and they are all powered by batteries. Some researchers have proposed many routing protocols for energy-efficiency in WSNs [13,14]. Therefore, green communication has extensive applications. Many previous studies have investigated green communication, the DCMST algorithm, and GAs to reduce network energy consumption.

### 2.1. DCMST Methods

The minimum spanning tree (MST) is a classic topological technique used in transportation, communication, and other fields. This technique is mainly used to solve the shortest path problem in the case of full connectivity. Many network optimization and operational research problems have been solved based on the MST. Specifically, with the application of MSTs in many fields, it has become an increasingly popular research topic, and many effective methods have been proposed. The MST algorithm was first formulated in [15], with many additional contributions in the early 20th century. The MST was first used in electrical network planning. An example of an electrical network that served 40 cities was proposed is the first application of the MST algorithm [16]. However, due to differing requirements, the MST approach is often limited in solving practical problems in different research fields.

In many studies, a more effective algorithm is the DCMST. The DCMST technique has been widely applied to optimize transportation networks, electrical wire networks and other networks in which the lengths of the connections between nodes should be minimized [17]. Degree constraints (which are a spanning tree) are based on the constraint values of nodes and the smallest weight. If a node is out of commission, the degree constraint mechanism is used to prevent the node from becoming invalid. The DCMST algorithm can be introduced to establish a network with the maximum number of crossing paths.

The DCMST method is NP-hard [18], and it is based on network searching. A randomized primal method (which is a novel tree construction algorithm) was proposed and used to establish a low-overhead degree-constrained tree for stochastic iterative search techniques and builds [19]. Network design problems were proposed to represent spanning trees with sets of edges. Based on black-box optimization techniques, an encoding method was used to initialize a tree [20]. However, the algorithm for a single tree is not applicable as the complexity of the network grows. Node-depth encoding was proposed for forest representation. This approach is based on the union of the encodings of all trees in a forest. The union includes an array of pointers, and each pointer comprises a tree consisting of linear lists containing the tree nodes and their depths [21]. A node-depth-degree representation was proposed to generate and manipulate a set of spanning forests [22]. This structure improved the average run time of previous node-depth algorithms.

### 2.2. Genetic Algorithm

Many studies have investigated heterogeneous networks based on GAs to reduce energy consumption and extend the lifetime of the network.

In the Stable Election Protocol (SEP) [23], cluster heads are elected based on weighted probabilities that depend on the residual energies of sensor nodes. Then, based on categorizing the energy levels of sensor nodes, the Developed Distributed Energy-Efficient Clustering (DDEEC) [24] method was introduced to improve the SEP. The nodes with high energy levels are the “advanced nodes”, and the cluster head is selected from these advanced nodes.

The Threshold Sensitive Stable Election Protocol (TSSEP) [25] also improved the original SEP. In this approach, the energy levels of mobile nodes are divided into three groups according to the threshold intervals of the cluster head node. In addition, cluster heads are elected based on the proportion of their residual energy according to the Energy-Efficient Heterogeneous Clustering (EEHC) scheme [26] and the Efficient Three-Level Energy algorithm (ETLE) [27]. ETLE established three stable levels of energy to characterize node heterogeneity. Cluster head nodes are chosen based on these standards and solve the effect of node energy heterogeneity on the network lifetime. In the Hybrid Energy-Efficient Reactive protocol (HEER) [28], a cluster head is elected based on the ratio of the residual energy of nodes to the average energy level of the network. It chosen the cluster head nodes by using APIT (Approximate Point-In-Triangulation) to measure the distance and the energy consumption functions of heterogeneous nodes and solves the impact of heterogeneous energy of nodes on clustering.

However, the clustering algorithms described above are only heterogeneous for the initial energy of the mobile node. Therefore, these algorithms are not suitable for a GCUM. The initial energy of mobile nodes differs, and the energy consumed by transmission varies due to differences in functionality. In other algorithms, connectivity also influences whether a mobile node is selected as a cluster head node, and the number of neighbor nodes around a mobile node affects connectivity. For example, a mobile node with many neighbor nodes is more likely to become a cluster head node. Low mobile node connectivity often means that the transmission of information is limited. Therefore, poorly-connected nodes are less likely to become cluster head nodes. When the clustering process begins, each mobile node begins to update its neighbor table. It concurrently broadcasts packets to the neighbor nodes and receives packets, thereby determining its own connectivity during this process. In [29], a cluster head node was elected primarily based on the degree of connectivity. In addition to the degree of connectivity of a node (i.e., the density of nodes), the number of neighbors is also used as a research criterion. Intuitively, if a sensor has more neighbors, it will have a higher probability of serving as a cluster head node. At the start of the clustering process, each node receives information from its neighborhood through broadcasted hello packages. In the Energy-Efficient Unequal Clustering (EEUC) method [30], the energy consumption of nodes, before clustering, is considered to determine the optimal cluster size. A two-stage GA is employed to determine the optimal interval of cluster size and derive the exact value from the interval. The proposed Circular Motion of Mobile-Sink with Varied Velocity Algorithm (CM2SV2) balances the energy consumption ratio of cluster heads.

These algorithms often focus on a single factor. Additionally, many intelligent algorithms use routing protocols based on clustering algorithms that can be applied to solve enhanced learning algorithms, ant colony algorithms, fuzzy logic algorithms, and GAs that are similar in highly complex and dynamic environments with multiple factors. In addition, many studies have proposed various clustering mechanisms, such as the Local Negotiated Clustering Algorithm (LNCA) [31]. The LNCA is a new clustering method in which the clustering process is based on the similarity of mobile nodes. The Evolutionary-based Clustered Routing Protocol (ERP) [32] overcomes the limitations of GA-based clustering algorithms by combining the clustering aspects of cohesion and separation error, and it implements a new fitness function based on these two factors. The dynamic clustering of heterogeneous wireless sensor networks based on a GA (DCHGA) [33] improved the ERP. It considered many heterogeneous factors, e.g., the data processing capability of different nodes, and the ability to serve as a cluster head. The GA is also used to obtain an optimal clustering result and extended the lifetime of the network. In this approach, information is dynamically updated after each transmission round. Additionally, several factors are integrated based on the framework. However, the DCHGA method has no clear route for assessing these heterogeneous factors.

In this paper, both the DCMST and GA are introduced to assess the relevant heterogeneous factors. The initial clustering process is based on this information.

## 3. GCUM and the Energy Consumption Model

In this section, the three models of anchor, multifunction and original nodes are defined. The nodal models and energy consumption functions are proposed.

### 3.1. GCUM Model

(1) The structure of the GCUM includes many tunnels that cross and create a complex network environment.
(2) In the clustering process, anchor nodes are chosen based on specific priorities, and cluster head nodes are selected. However, when special circumstances, such as accidents, occur, if the original anchor nodes are damaged, mobile nodes with high residual energy demands may become the new cluster head nodes.
(3) The three types of nodes are anchor, multifunction, and original nodes. Each node has a different initial energy level, and their functions are influenced by the heterogeneous factors of the GCUM.

### 3.2. Nodes in the GCUM

In this paper, the different mobile nodes are reflected in the GCUM. These three types of nodes are anchor nodes (ANs), ordinary nodes (ONs), and multifunction nodes (MNs).

ANs have wired power supplies fixed on the tunnel walls in underground mines. The sensors on these nodes are used to collect environmental information, such as temperature, humidity, and gas concentration data. If the power wires of these nodes are damaged in accidents, their power supplies switch to batteries. However, to achieve green communication, the energy consumption of anchor nodes must be considered, even for wired power supplies. ONs are deployed on the miners with localization and emergency calling functions and are powered by batteries. Under normal working conditions, ONs only receive, send, or forward packages with localization information. In the case of an emergency, a miner could press the call button to send a distress package to upper-level systems. MNs include all the functions of ONs and have many additional functions, including environmental status monitoring and camera, audio, and video functionality. Therefore, upper-level systems can monitor the real-time status of underground mining in a variety of ways, such as via photographs, audio or videos. Furthermore, if the initial energy of ONs is limited, video and audio data are not forwarded by ONs to reduce energy consumption. Video and audio data are uploaded to upper-level systems by ANs only.

### 3.3. Energy Consumption Model

The GCUM is a two-dimensional space, data are propagated in the clustering process in the following ways [34]:

◆ $E_{s}^{A}\left(\lambda_{1}t\right)$ represents the energy consumption of acquiring $\lambda_{1}t$ *bits* data.
◆ $E_{s}^{P}\left(\lambda_{2}t\right)$ represents the energy consumption of processing $\lambda_{2}t$ *bits* data.
◆ $E_{s}^{T}\left(\lambda_{2}t,d\right)$ represents the energy consumption of transmitting $\lambda_{2}t$ *bits* data over distance *d*.
◆ $E_{s}^{R}\left(\lambda_{3}t\right)$ represents the energy consumption of cluster head receiving $\lambda_{3}$ *bits* data from its member nodes.

Where $\lambda_{1}$ denotes the rate of acquiring data, $\lambda_{2}$ denotes the rate of processing and transmitting data, $\lambda_{3}$ denotes the rate of cluster head receiving data from its member nodes, and t is the time represented by the transmission rounds. In addition, $E_{s}^{T}\left(\lambda_{2}t,d\right)$ and $E_{s}^{R}\left(\lambda_{3}t\right)$ can be described in detail as following functions:

$$E_{s}^{T}\left(\lambda_{2}t,d\right)=E_{i}\left(t\right)+\lambda_{2}td^{m}$$

$$E_{s}^{R}\left(\lambda_{3}t\right)=E_{i}\left(t\right)+\lambda_{3}tE^{\prime }$$

where $E_{i}$ indicates the energy consumption of idle process. d indicates the communication distance between the two connected nodes, m is the energy consumption parameter of the transmitting distance, m=2 is defined as the short distance of transmitting within the radius range of the cluster head, and m=4 is defined as the long distance of transmitting data to the receiving node with multi-hops. $E^{\prime }$ indicates the overhead of the beam forming to decrease the energy consumption. Therefore, the energy consumption function can be established as follows:

(1) $$E_{s}\left(t\right)=E_{s}^{A}\left(\lambda_{1}t\right)+E_{s}^{P}\left(\lambda_{2}t\right)+E_{s}^{T}\left(\lambda_{2}t,d\right)+E_{s}^{R}\left(\lambda_{3}t\right)$$

Thus, the residual energy of node s can be written as:

(2) $${\hat{E}}_{s}\left(t\right)=E_{s}\left(0\right)-\overset{t}{\underset{i=1}{\sum }}E_{s}\left(i\right)$$

where $E_{s}\left(0\right)$ represents the initial energy of node s at time t, $\lambda_{1}$ represents the generation rate of data in the network due to the status of network, $\lambda_{2}$ represents the throughput of data in channels or nodes, and $\lambda_{3}$ represents the receiving data of member nodes decided by the number of member nodes.

In addition, the energy consumption in the cluster will be also considered. The energy consumption of member nodes and cluster head nodes, as below:

(3) $$\begin{array}{c} {\tilde{E}}_{s^{\prime }}=E+\lambda_{2}tD^{2}\left(s^{\prime },s\right)\\ {\tilde{E}}_{s}=E+N_{s}\lambda_{2}tE^{\prime } \end{array}$$

where E denotes the inherent energy consumption of nodes by acquiring, processing and idle. $s^{\prime }$ and s denotes member nodes and cluster head nodes, and $D^{2}\left(s^{\prime },s\right)$ denotes the distance between member nodes and the cluster head node.

In this paper, throughput $\lambda_{2}$ can be calculated by the effective can be expressed as follows:

(4) $$\lambda_{2}=\frac{1}{V\lambda_{1}}\overset{\left|V\right|}{\underset{z=0}{\sum }}Q_{z}\left[z\frac{\left(1-{\left(1-P_{a}\right)}^{K}\right)L}{T_{s}}\right]$$

where V indicates the set of the node in the network and |V| indicates the number of nodes in this set, $Q_{z}$ indicates that there are z nodes need to transmit data, $1/T_{s}$ is the transmission rate of data packets, *K* is the retreat time upper limit and $P_{a}$ is the probability of a channel being available. Specifically, $Q_{z}$ and $P_{a}$ can be formulated as follows:

(5) $$Q_{z}=\frac{\left|V\right|!}{z!}p^{z}$$

(6) $$P_{a}=P_{a|a}P_{a}+P_{a|b}\left(1-P_{a}\right)$$

where $P_{a|a}$ and $P_{a|b}$ are conditional probability of channel availability based on whether the channel was available or not at the previous moment, and p is the probability that the data needs to be sent.

## 4. Routing Algorithm Based on DCMST

**Figure 1:**
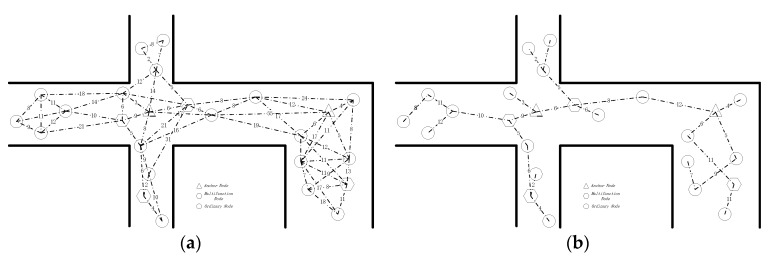
(**a**) Diagram of a node communication network in an underground mine. (**b**) Diagram after the DCMST is applied to the node communication network in an underground mine.

GCUM functions are used to manage miners who are wearing the mobile node modules, supervise the production schedule under normal conditions, and protect miners in the mine when accidents occur. Reliable connectivity, as established from the DCMST, ensures that nodes in the network are connected, and an optimized network structure can be obtained in which energy consumption is reduced and the network lifetime is extended. Therefore, the routing algorithm based on the DCMST can effectively distinguish among node types. In the context of low power consumption and green communication, the DCMST can identify reliable routing paths and derive the weight equation of each path and the degree of residual energy of each node. Figure 1a shows a diagram of the communication between nodes in tunnels in an underground mine. The numbers that are marked on the lines are the weights of the respective routing paths. The DCMST is introduced to initialize the MST of the network. Each edge in the MST is a routing path between connected nodes that guarantees connectivity and achieves the lowest overhead, as shown in Figure 1b.

In this paper, the GCUM is regarded as a simple, connected, and undirected graph, and a DCMST algorithm for heterogeneous nodes is proposed [35]. *G* is an undirected complete network; *V* is the set of nodes in the GCUM; and *E* is the set of edges. Additionally, $W={\left(w_{ij}\right)}_{n\times n}$ is the weight matrix of G, where $w_{ij}=w_{ji}\;\mathrm{and}\;w_{ii}=+\infty \;\left(i,\;j=1,2,\ldots ,\;n\right)$. If $v_{i}v_{j}\in E,\;\mathrm{then}\;w_{ij}=w\left(v_{i}v_{j}\right)$; otherwise, $w_{ij}=+\infty$. Moreover, $h_{i}\;\left(i=1,\;2,\;\ldots ,n\right)$ is defined as the degree of each node in the network.

**Definition 1.**  G(V,E,W)*is defined as an undirected complete network*, *where*$v_{0}\in V$*is a node of* G *and* y *is a positive integer*. *If T is a spanning tree of G and* $d_{t}\left(v_{0}\right)=y$, *T is the h-degree constraint spanning tree for node* $v_{0}$ in network G.

**Definition 2.**  G(V,E,W)*is defined as an undirected complete network*, *where*$v_{0}\in V$*is a node of G*. *Among all spanning trees of G*, *if the degree k of*$v_{0}$ is maximized and the weights of the edges are minimized in the spanning tree, the tree is defined as the maximum degree constraint MST of node $v_{0}$ *in G*.

Therefore, the weight of edges can be replaced by $E_{s}^{T}\left(\lambda_{2}t,d\right)$, and searching for the minimum weight of an edge is equivalent to find the minimum $E_{s}^{T}$ with minimum parameters of throughput $1-\lambda_{2}$ and distance d.

In addition, $d_{T^{\prime }}\left(v_{0}\right)$ will be defined as the degree of node $v_{0}$, which represents the maximum edges that connect with it. In this paper, the degree of the nodes will be constrained to prevent unnecessary energy consumption by multi-path connections. Therefore, according to the residual energy function (Equation (2)), two thresholds, $\delta_{0}$ and $\delta_{1}$ ($0<\delta_{0}<\delta_{1}<\;\mathrm{initial}\;\mathrm{energy}$), are set to assign the degree h. The maximum degree of ONs is set to 2. When the residual energy is above $\delta_{1}$, the maximum degree of ANs and MNs are set as unlimited and six, respectively. When the residual energy is interposed between $\delta_{0}$ and $\delta_{1}$, the maximum degree of ANs and MNs are set as six and four, respectively. When the residual energy is below $\delta_{0}$, the maximum degree of ANs and MNs are both set to two. Constraining the degree of the node effectively controls the multi-path connection problem and effectively extends the network lifetime.

Therefore, G(V,E,W) is defined as an undirected complete network, where $v_{0}\in V$ is a node of *G*. A maximum degree constraint $T^{\prime }$ must exist for node $v_{0}$ of *G*, and $h_{T^{\prime }}\left(v_{0}\right)=h_{G}\left(v_{0}\right)$. $h_{T^{\prime }}\left(v_{0}\right)$ is the degree of $v_{0}$ in $T^{\prime }$, and $h\left(v_{0}\right)$ is the degree of $v_{0}$ in *G*.

The DCMST model is as follows:

(7) $$minW=\overset{n}{\underset{i=1}{\sum }}\overset{n}{\underset{j=1}{\sum }}w_{ij}\tau_{ij}$$

(8) $$\{\begin{array}{c} \overset{n}{\underset{j=1}{\sum }}\tau_{ij}\leq h_{i},\;i\in V\\ \underset{i,j\in S}{\sum }\tau_{ij}\leq \left|S\right|-1,\;\forall S\in V,\;S\neq \emptyset \\ \overset{n}{\underset{i=1}{\sum }}\overset{n}{\underset{j=1}{\sum }}\tau_{ij}=n-1\\ \tau_{ij}\in \left\{0,1\right\},\;i,\;j\in V \end{array}$$

If edge *(i*, *j)* is in the optimal tree, $\tau_{ij}$ = 1; otherwise, $\tau_{ij}$ = 0. |S| is the number of nodes in *G* that are contained in the set S.

The steps of the DCMST Algorithm 1 are as follows:
**Algorithm 1.** DCMST algorithm.**Input:**V: Set of nodes; $v_{k}$: Nodes; $V_{G}\left(v_{k}\right):$ Set of nodes in tree *G*; $E_{G}\left(v_{k}\right)$: Set of edges in tree *G*;**Output:**$T_{k}$: DCMST of the network$G\leftarrow \left|V\right|-\left|V_{G}\left(v_{k}\right)\right|-1$$V_{G}\left(v_{k}\right)=E_{G}\left(v_{k}\right)=\emptyset$$S_{0}=\left\{v_{0}\right\}\cup V_{G}\left(v_{0}\right)$$E_{0}=E_{G}\left(v_{0}\right)$$S_{0}^{\prime }=\frac{V}{\left\{v_{0}\right\}}\cup V_{G}\left(v_{0}\right)$**For**k=1; k≤|V|**do**  **While** $v_{k}$ neighbored $v_{0}$ **do**
   $V_{G}\left(v_{k}\right)=V_{G}\left(v_{k}\right)\cup V_{G}\left(v_{0}\right)$  **EndWhile**  **While** $v_{k}$ associated $v_{0}$ **do**
   $E_{G}\left(v_{k}\right)=E_{G}\left(v_{k}\right)\cup E_{G}\left(v_{0}\right)$  **EndWhile**  $S_{k:}=S_{k-1}\cup \left\{v_{k-1}^{\prime }\right\}$  $E_{k:}=E_{k-1}\cup \left\{e_{k-1}\right\}$  $S_{k}^{\prime }=\frac{S_{k-1}^{\prime }}{\left\{v_{k-1}^{\prime }\right\}}$  **If** $S_{k}=V$ **then**   $T_{k}=\left(S_{k-1},\;E_{k-1}\right)$  **Else** $w\left(e_{k}\right)=\underset{e\in \left[O_{k},\;O_{k}^{\prime }\right]}{\min}w\left(e\right)$, $e_{k}=v_{k}v_{k}^{\prime }$  **EndIf**  **If** $\left[S_{k},\;S_{k}^{\prime }\right]=\emptyset$ **then**   $T_{k}=\emptyset$  **EndIf****EndFor**

Finally, $T_{k}$ is the maximum degree constraint minimum degree spanning tree of the GCUM. Based on the edges that could be equivalent to routing paths, the clustering structure is introduced in the GCUM to reduce the energy consumption of the network and improve its longevity. In Section 4, minimizing the weights of routing paths based on the DCMST can effectively reduce consumption of energy of the transmission and increase the throughput rate in the network to achieve the goal of green communication.

## 5. GA-Based Network Clustering Model

In the GCUM, because of the heterogeneity of initial energies, their functions, and the energy consumption levels of three types of nodes, a GA can be used to optimize the clustering results.

### 5.1. GA Definition

In the conventional state, the ANs initiate the clustering process as the cluster head node, and the MNs and ONs are involved in the clustering process as the cluster member nodes. However, in some special cases (such as during underground mining accidents), the structure of the underground mine changes. Specifically, some anchor nodes may be damaged and cannot serve as cluster head nodes. Therefore, in this case, the proposed GA established the rules for multiple functions, and ordinary nodes serve as cluster head nodes. Since there is no integration, the mechanisms in traditional network clustering algorithms cannot be applied to a heterogeneous environment. Therefore, in the GCUM in this paper, the network model must be optimized before using the GA chromosome factor to optimize the network clustering structure.

The characteristics of the nodes in the network are analyzed in the process of network structure optimization based on the GA. The chromosomes of the GA are defined as the cluster structure of the GCUM, and genes are defined as the nodes of the cluster. Genes are classified and assigned based on the GA and serve as cluster heads or member nodes according to their values. Two variable lists are introduced to record the status of each node. One list is the residual energy, and the other is the role. In the residual energy list, if the value of a node equals one, the node has sufficient residual energy, and is a cluster head node candidate. If the value equals zero, the node does not have sufficient residual energy and cannot serve as a cluster head node. In the role list, if the value of a node equals 1, the node has served as a cluster head node. If the value equals 0, the node has served as a member node. If a node has sufficient residual energy and can be a cluster head node, it will be included in subsequent calculations.

To control the number of individuals and avoid clustering results converging to a local optimum, the GA generates new chromosomes through crossover and mutation processes.

(1) The crossover process randomly chooses two individuals to exchange certain information at a high level of probability. The purpose of the exchange process is to generate a new genetic combination to limit the loss of genetic material. The generation and evolution rate of new chromosomes can be controlled by changing the crossover probability. If the crossover process does not occur, the parent chromosome that is ready to participate in the crossover process will be directly included in the subsequent calculations without any changes.
(2) The mutation process changes some of the population at a lower probability than that associated with the crossover process. A gene on a chromosome is randomly selected, and its assignment is changed with the goal of preventing premature convergence during the optimization process. Unlike the crossover process, the mutation process can be controlled by changing the mutation probability, and new individuals can be produced in this process to reach a local optimum. When a node fails, its corresponding gene assignment will change, and it will no longer be included in the subsequent mutation processes.

### 5.2. Establishment of a Fitness Function

Based on the DCMST algorithm, the optimal cluster structure required to minimize the weights W of all the routing paths from a chromosome perspective is as follows:

(9) $$W^{*}=\overset{C}{\underset{i=1}{\sum }}\overset{M_{v_{i}}}{\underset{j=1}{\sum }}W\left(v,v_{j}\right)$$

where *C* is the number of clusters in the GCUM and $M_{v_{i}}$ is the number of member nodes in a cluster with cluster head node $v_{i}$.

Therefore, the residual energy, weight, and density of each node will be employed in the fitness function as follows:

(10) $$f=\underset{s}{\sum }\frac{\hat{E_{s}}\left(t\right)}{E_{s}\left(0\right)}+\frac{\hat{E_{s}}\left(t\right)}{{\tilde{E}}_{s^{\prime }}+{\tilde{E}}_{s}}+\frac{1}{W^{*}}$$

This fitness function is used to evaluate the fitness of chromosomes. A medium library that can be customized depending on the specific GCUM is established to control the individuals created in a generation. When the calculations reach the maximum number of steps, or the fitness function converges, the cluster structure of the GCUM is established.

### 5.3. GA Processing

Some parameters must be defined in the proposed algorithm. *Q* and *q* represent the size and number of generations, respectively. *P* denotes the population size. *L* indicates the library of chromosomes of individuals that are randomly generated. *n* indicates the number of nodes.

In the crossover process, based on the crossover probability, two chromosomes are randomly chosen from *L*, and two new chromosomes are generated by switching consecutive genes. In the mutation process, based on the mutation probability, a gene is randomly chosen, and its value is set between zero and one.

Nodes in the GCUM are represented by a chromosome library *K*, where $K=\left\{k_{1},\;k_{2},\ldots ,k_{n}\right\}$. Based on function (6) and the routing paths from the DCMST, the nodes in the network are clustered. The fitness of each node is calculated by function (7). A normalized fitness function is introduced as follows:

(11) $$\tilde{f}\left(k\right)=\frac{f\left(k\right)}{\sum_{c}f\left(k\right)}$$

Two different chromosomes are randomly chosen from K. Based on the crossover probability, α is converted to $k_{a}^{\prime }$ and $k_{b}^{\prime }$ by the crossover process. Then, based on the mutation probability *β*, $k_{a}^{\prime }$ and $k_{b}^{\prime }$ are changed to $k_{a}^{*}$ and $k_{b}^{*}$ by the mutation process. Based on the new fitness level, $f\left(k_{a}^{*}\right)$ and $f\left(k_{b}^{*}\right)$, which are calculated based on $k_{a}^{*}$ and $k_{b}^{*}$, a restructured function is introduced to obtain the clustering structure based on the new library of chromosomes:

(12) $$k^{*}=arg\underset{k}{\max}f\left(k\right)$$

Therefore, a new cluster structure is obtained by the GCUM when the calculation times reach *Q* or the calculation converges.

## 6. Simulation Results and Analysis

In this paper, to achieve the goal of green communication, an effective method is established to balance the network load and energy consumption of mobile nodes in the network. After a certain number of rounds, the energy consumption of nodes in the network is balanced to prevent some nodes from prematurely failing, which negatively affects the lifetime of the entire network. To achieve this goal, simulation experiments involving the GCUM were conducted using the MATLAB (MathWorks, Natick, MA, USA) simulation platform by a desktop computer with Intel Core i5-7500 @ 3.40 GHz Quad Core, 8 GB DDR4 2400 MHz memory, and the Windows 7 operating system. The ORAGC has two steps of calculation with DCMST and GA. Thus, the time complexity *O* is the sum of these two algorithms described as:

$$O=O\left(E*\mathrm{lg}E\right)+O\left(n^{2}\right)$$

where *E* is the number of edges in the DCMST and *n* is the number of nodes in the network.

The environmental parameters and structure were defined as follows.

(1) The experimental area is simulated as tunnels in the GCUM, in which there are long passages and intersections. Anchor nodes are deployed with the same interval distance of 50 m in tunnels.
(2) There are two types of mobile nodes in the GCUM: ordinary nodes and multifunction nodes. Multifunction nodes comprise 10% of the total number of mobile nodes in the network.
(3) In a normal scenario, anchor nodes serve as cluster head nodes when the cluster process is initiated. In a special case, such as an accident, mobile nodes, including both ordinary and multifunction nodes, can replace anchor nodes as cluster head nodes.
(4) In the GA algorithm, the population size is 30. The performance of the final optimization may be negatively impacted by a high probability of mutation. To compare fairly in the later experiments, we used the same probability of mutation and crossover as DCHGA and other algorithms. Thus, the mutation probability β is set to 0.006. In addition, the convergence results will exhibit better correlation at low probabilities of crossover. Thus, the crossover probability α is set to 0.8.
(5) The transmission ranges of anchor, multifunction and ordinary nodes are 50 m, 30 m and 15 m, respectively.

The simulation parameters are set by 100 mobile nodes, including 10 multifunction nodes and 90 ordinary nodes, which are deployed in the simulation area. Respectively, the initial energy of ANs, MNs, and ONs is set as 10 J, 1.5 J, and 0.5 J, and the energy consumption at idle is 50 nJ/bit. In addition, packet size is set as 300 bits, and thresholds $\delta_{0}$ and $\delta_{1}$ are set as 0.4 J and 1 J. Table 1 shows the average processing time of ORAGC.

**Figure 2:**
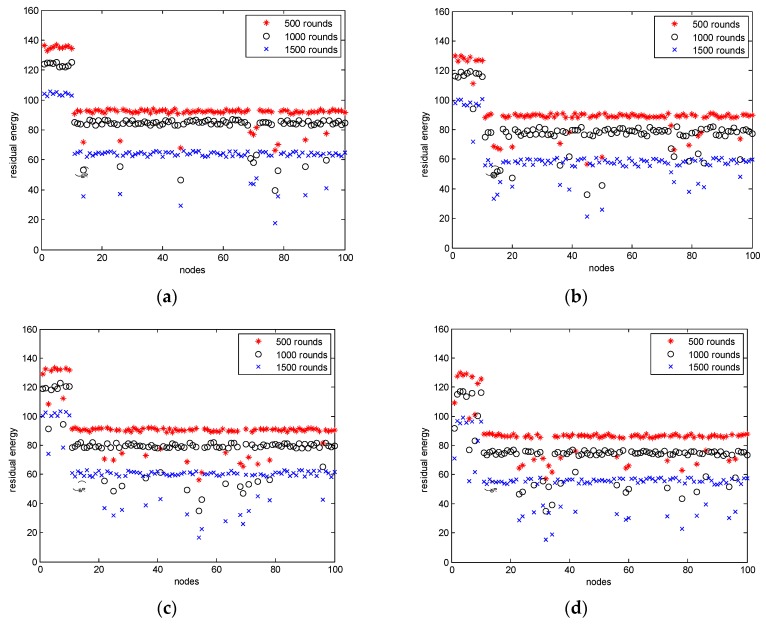
(**a**) Residual energy in different rounds in scenario 1. (**b**) Residual energy in different rounds in scenario 2. (**c**) Residual energy in different rounds in scenario 3. (**d**) Residual energy in different rounds in scenario 4.

Figure 2a shows scenario 1 in a long tunnel without intersections where anchor nodes are deployed at intervals of 50 m. Figure 2b shows scenario 2 in a long tunnel without intersections where anchor nodes are deployed at intervals of 200 m to simulate that some anchor nodes were damaged in an accident. Figure 2c shows scenario 3 in an area of tunnels with a large number of intersections where anchor nodes are deployed at intervals of 50 m. Figure 2d illustrates scenario 4 in an area of tunnels with a large number of intersections where anchor nodes are deployed at intervals of 200 m to simulate that some anchor nodes were damaged in an accident. As shown in these figures, the algorithm performance is best for scenario 1 because the network comprises long tunnels without blockages and transmission occurs based on line of sight (LoS). In scenario 2, due to the presence of intersections, some transmissions cannot be directly processed, and multihops that are forwarded by certain mobile nodes must be used. This process results in higher energy consumption. Therefore, in scenarios 3 and 4, more energy consumption is required because LoS exists and fewer anchor nodes are available. In general, these conditions will increase energy consumption in GCUMs.

In this paper, five routing algorithms (ETLE [27], HEER [28], EEUC [30], ERP [32], and DCHGA [33]) used in the network clustering process are introduced for comparison with the optimization routing algorithm for green communication (ORAGC) in underground mines proposed in this paper.

**Figure 3:**
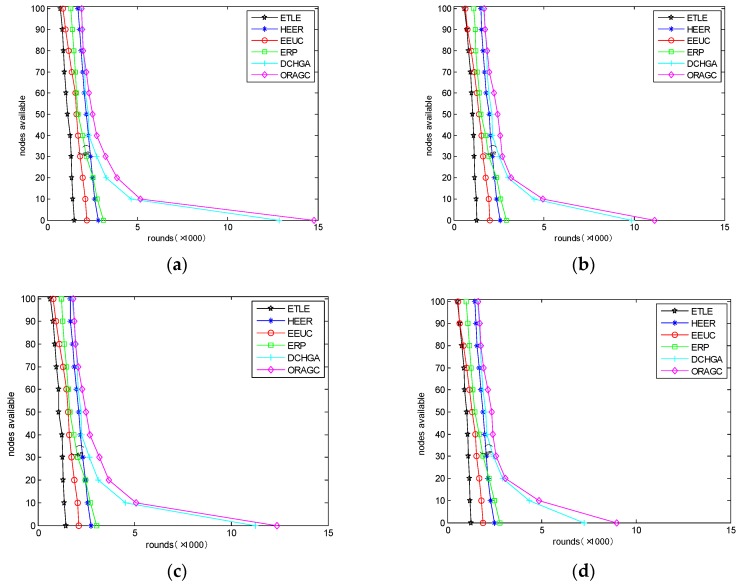
(**a**) Energy consumption of the routing algorithms in scenario 1. (**b**) Energy consumption of the routing algorithms in scenario 2. (**c**) Energy consumption of the routing algorithms in scenario 3. (**d**) Energy consumption of the routing algorithms in scenario 4.

The comparison of lifetimes is shown in Figure 3. Notably, the ORAGC algorithm saves the most energy in the four different scenarios (as previously described). This result occurs because some routing algorithms, such as the ETLE and ERP methods, cannot address heterogeneous factors, and only one factor is considered in these algorithms. For example, the heterogeneity of the initial energy is the only factor considered in the ETLE. Therefore, if the network connectivity is poor, the collision rate will be high and result in a high probability that retransmission will cause extra energy consumption, and vice versa. The DCHGA considers different heterogeneous factors in the clustering process, but the energy consumption model is not optimized for the heterogeneity of the network. Therefore, the ORAGC improves this approach, and a weight model is established that considers connectivity, energy consumption, and delays based on the DCMST.

The following tables list the rounds when the first and last nodes failed in the network. In comparing the data, the network lifetime can be effectively assessed.

To verify the performance of the ORAGC, the five routing algorithms are compared based on the network lifetimes estimated by the simulation experiment. The results in Table 2 and Table 3 show that the ORAGC yields the best performance in the four different scenarios. In scenario 1, the first node fails after 1910 rounds, and the last node fails after 14,780 rounds based on the ORAGC. These results are 7.3% and 15.02% better than those of the DCHGA, respectively. In scenario 2, the first node fails after 1690 rounds, and last node fails after 11,130 rounds for the ORAGC. These results are 8.33% and 12.99% better than those of the DCHGA, respectively. In scenario 3, the first node fails after 1830 rounds, and last node fails after 11,130 rounds for the ORAGC. These results are 8.93% and 10.24% better than those of the DCHGA, respectively. In scenario 4, the first node fails after 1640 rounds, and last node fails after 8960 rounds for the ORAGC. These results are 8.93% and 10.24% better than those of the DCHGA, respectively. In addition, the lack of anchor nodes has a larger impact on the network lifetime than does the number of intersections (or lack of LoS). In general, the ORAGC considers the heterogeneous factors in the network and establishes an energy consumption model and routing paths based on the DCMST to initialize cluster processing based on the GA. The results show that the ORAGC exhibited better performance than other routing algorithms for the given GCUM.

## 7. Conclusions

Green communication is introduced as an advanced approach to underground mine WSNs, which are comprised of numerous nodes with heterogeneous initial energies, data processing capabilities, and functions. The ORAGC is proposed in this paper to achieve the goals, including saving energy, satisfying the requirements of extending the network’s lifespan, and function implementation. To adapt to a variety of heterogeneous factors in the network, weight and routing paths are concurrently introduced based on the DCMST. Then, an energy consumption model is established with many heterogeneous factors in the network. Based on the former routing paths, the GA is introduced to initialize the clustering process and balance the energy consumption and longevity of the network. Since different heterogeneous factors are considered, nodes in the network must be identified to create an optimized routing path with a low collision rate and low energy consumption. The ORAGC improved the network lifetime by 7.3% to 8.93% based on the failure of the first node and 15.02% to 23.93% according to the failure of the last node.

## Figures and Tables

**Table 1:** Average processing time of ORAGC with 500 rounds by increasing the number of nodes in the network.

Number of Nodes	10 MNs and 90 ONs	15 MNs and 135 ONs	20 MNs and 180 ONs	25 MNs and 225 ONs
Average processing time (/ms)	468	1029	1697	2681

**Table 2:** Running rounds when the first node fails of six algorithms in four different scenarios, and the improvement is given.

Rounds When the First Node Fails (Rounds)				
Techniques	Scenario 1	Scenario 2	Scenario 3	Scenario 4
ETLE	730	580	640	520
HEER	1720	1520	1670	1450
EEUC	870	640	780	570
ERP	1310	1120	1220	990
DCHGA	1780	1560	1680	1510
ORAGC	1910	1690	1830	1640
Improvement	7.30%	8.33%	8.93%	8.61%

**Table 3:** Running rounds when the last node fails of six algorithms in four different scenarios, and the improvement is given.

Rounds When the Last Node Fails (Rounds)				
Techniques	Scenario 1	Scenario 2	Scenario 3	Scenario 4
ETLE	1520	1290	1440	1240
HEER	2850	2590	2740	2490
EEUC	2190	2010	2120	1900
ERP	3120	2930	3030	2780
DCHGA	12,850	9850	11,230	7230
ORAGC	14,780	11,130	12,380	8960
Improvement	15.02%	12.99%	10.24%	23.93%
